# Analysis on determinants of carbon emissions from plaza ground paving during the construction stage based on life cycle assessment

**DOI:** 10.1038/s41598-023-47933-9

**Published:** 2024-01-02

**Authors:** Huayue Nie, Lizhong Wang, Meirong Tian

**Affiliations:** 1 Chinese Research Academy of Environmental Sciences, Beijing, 100020 China; 2 College of Environmental Science and Engineering, Tongji University, Shanghai, 200082 China; 3 Beijing Orange Stone Technology Co., Ltd, Beijing, 100020 China

**Keywords:** Environmental impact, Energy and society, Sustainability

## Abstract

The carbon emissions of paving projects are the focus of urban managers and researchers. By introducing the life cycle assessment (LCA) method and drawing up the study time and boundary, this study analyzed the carbon emissions activities of the plaza ground paving project and established a computational model of the cast-in-place architectural concrete (CAC) and natural stone pavement’s life cycle during the construction stage by comprehensively utilizing the carbon emission coefficient method and the direct source consumption statistics method of the production line. Based on the model, this study employed the ground paving of a top-notch Theme Park Plaza in Beijing as a sample to calculate the carbon emissions of two different types of building materials at various phases of their life cycle and made a comparative evaluation. It is concluded that the carbon emissions (expressed in CO_2_) produced by the CAC ground in the sample area is 75.46 kg CO_2_/m^2^, while that of the natural stone pavement is 110.81 kg CO_2_/m^2^. Our results demonstrate significantly linear relationship between the overall emissions of carbon and the material carbon factor. This study adds to the body of knowledge by calculating the carbon emissions and determining the trend of carbon footprint for ground paving. Furthermore, the study's findings can be used to enhance construction management options and choose green materials. The findings can also be used to provide supporting theories for the development of regulations and carbon reduction policies based on constructing energy conservation and greenhouse gas reduction.

## Introduction

The greenhouse effect has become a widespread issue faced by the whole world and has a significant impact on human survival and progress^1^. “Carbon emissions” is an abbreviation for greenhouse gas emissions. Since CO_2_ is the most significant greenhouse gas, the word “carbon” is used as a representative, and “carbon emissions” is also understood as “CO_2_ emissions”^2,3^. The increase in carbon emissions will have a serious impact on social and economic development, which has attracted the attention of all countries in the world^4^. According to the research from World Business Council for Sustainable Development (WBCSD), in today's energy consumption structure, building energy consumption accounts for a large proportion, surpassing industrial energy consumption^5^. Statistics show that by 2020, carbon emissions from the construction sector accounted for 37% of all global emissions^6^. The three major sources of global carbon emissions are the construction, industrial, and transportation sectors^7^. Therefore, it is crucial to accurately assess buildings' carbon emissions and reduce their overall carbon footprint. SAP (Standard Assessment Procedure) issued by the Ministry of the Interior of the United Kingdom provided a comprehensive assessment of residential buildings as well as a calculation method for estimating the energy consumption of buildings an took the environmental impact grade of CO_2_ as a vital assessment index^8^.

The life cycle assessment (LCA) process quantifies and identifies the use of substances and energy in products, manufacturing processes, and activities, as well as their environmental emissions^9^. LCA covers the whole life cycle of commodities, production processes, and activities, including raw material exploration and processing, manufacturing, and transporting, as well as the use, reuse, maintenance, recycling, and final treatment^10^. The life cycle theory has been the foundation of extensive research into the estimation of the carbon emissions of construction engineering^11,12^. For instance, by measuring the carbon emissions of concrete based on the life cycle theory, Tae concluded that high-strength concrete consumes less energy and emits less CO_2_ than regular concrete^13^. Similarly, Zhao^8^ completed the carbon emission evaluation of the granite pavement in the garden using the life cycle theory, and Wu^14^ calculated the carbon emissions of recycled concrete pavement bricks across the whole life cycle. The LCA theory is also applied in the carbon emission calculation of residential buildings^15^, wind farms^16^, stone dams^17^, and other structures^18,19^.

The natural stone granite is chosen as the object in this study, mainly consisting of feldspar, quartz, and mica. Granite is resistant to acid, alkali, and weathering corrosion due to its hard texture. Moreover, granite also has a beautiful color that does not fade, which gives people a sense of nature. The excellent properties mentioned above make granite a popular material for outdoor ground decoration^20^. Architectural concrete is produced by mixing cement and pigment with different ratios and then adding proper amounts of plasticizer, curing, and release agent to produce a finish that resembles natural stone^21^. Architectural concrete refers to the architectural concrete designed, mixed, and poured at the construction site. Architectural concrete is economical, durable, and environmentally friendly, and can be adjusted in color and shape as needed, making it a popular material in the construction of large-scale parks^22^.

Various studies have been using LCA to assess the carbon output of construction projects but there is not much research on the plaza paving of large amusement parks. Therefore, in order to analyze the environmental impact of the paving process of the plaza ground, it is necessary to developing a comprehensive model to quantify the carbon emission in plaza ground paving project. Because a clear life-cycle framework for carbon emissions allows more detailed analysis of how to weaken carbon emissions at each stage. By quantifying carbon emissions from different materials, designers can receive more professional references when designing low-carbon buildings. In this study, we will calculate the carbon emissions of ground paving by developing a model based on LCA and calculate the carbon emissions of two different materials using a theme park plaza in Beijing as an example. Meanwhile, explore the determining factors and emission reduction potential of carbon emissions from ground pavement projects. In this research work, firstly, a concise literature review performing constructions carbon footprint is calculated using LCA. Second, refine the system boundary of the life cycle and construct a computational model by analyzing the process of ground laying at the case site. Third, estimate and compare the carbon emissions produced by paving with CAC and granite materials. Finally, examine the factors that affect carbon footprint during ground paving and the potential for emission reduction. In general, the findings of this study can offer theoretical foundation and technical support for evaluating the effect of technologies for energy conservation and emission reduction.

## Methods

### Boundary determination of life cycle assessment

**Figure 1:**
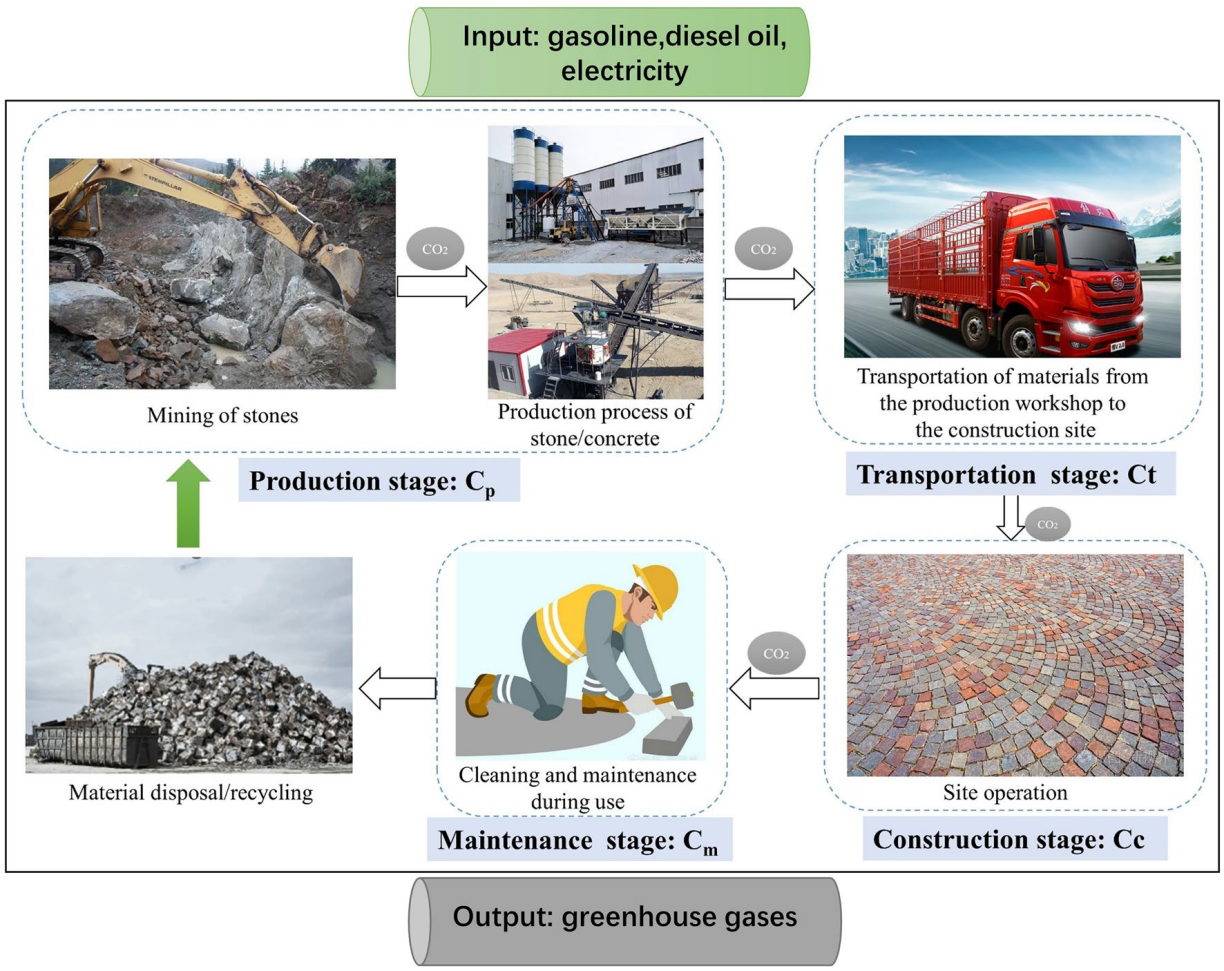
Life Cycle Framework of ground paving.

The life cycle framework of this study was built in accordance with “Life Cycle Assessment: Principles and Framework” (ISO 14040, 2006). In this study, the life cycle of the ground paving includes the production stage of materials (W_1_): the acquisition of raw materials, such as the mining of stones and the process of transporting the stones to the production workshop. Transportation stage (W_2_): carbon emissions produced from the fuel consumption of transporting raw materials from the origin to the paving site. Construction stage (W_3_): carbon emissions generated from the energy consumption of relevant construction machinery and equipment. Ground use and maintenance stage (W_4_): carbon emissions from pavement cleaning and maintenance. Cleaning and recycling stage (W_5_): carbon emissions generated from energy consumption of various mechanical vehicles. Considering that the carbon footprint is minimal because there is almost no machinery involved in the maintenance phase of the plaza, the production of raw materials is set as the starting point of the study time, with the formation of the ground paving as the endpoint (as shown in Fig. 1). Based on this, this study calculates the carbon emissions of the ground paving within the boundaries of this study time. The calculation formula of carbon emissions over the life cycle (during the project implementation phase) of a ground paving project is C_T_ = C_p_ + C_t_ + C_c_. (Cp, C_t_, C_c_ is the amount of carbon emission emitted by the production stage, transportation stage, construction stage, respectively). As an amusement park, almost no vehicles enter the square, and no machinery for maintenance and cleaning is required. The same carbon emissions during the maintenance phase have no impact on the comparison of the two materials. So, Carbon emission in the cleaning and maintenance stage is ignored.

### Selection of carbon emission factors

According to the research of the United Nations Intergovernmental Panel on Climate Change (IPCC) and other relevant institutions, in the statistical process on the production and use of materials, the relevant data of engineering quantities or energy consumption cannot directly reflect the carbon emissions produced by the corresponding activities^23^. In the case of analyzing the carbon emissions in actual construction projects, the carbon emission factor is introduced to convert different types of statistical data into carbon emission values. The amount of CO_2_ emitted is usually measured in terms of operability and economy^24^.

### Carbon emissions accounting model

In this study, a combination of the carbon emission coefficient method and the direct energy consumption statistics method of the production line was employed to calculate the carbon emissions. The direct energy consumption statistics method of the production line is to analyze and calculate the carbon dioxide emissions from the output and energy consumption data of the enterprise, which is used in the calculation of carbon emissions generated in the transportation, construction stage in this study. The calculation formula for carbon emissions generated by energy consumption is as follows:

1 $${\text{Q}}_{\text{1}} = {\text{P}}_{\text{1}}\times {\text{M}}_{\text{1}}\times {\text{I}}_{\text{1}}$$

where Q_1_ refers to the amount of carbon dioxide emitted by energy; P_1_ refers to the energy consumption per unit output; I_1_ refers to the emission coefficient of carbon dioxide emitted per unit of energy; M_1_ refers to the output.

The calculation formula for carbon dioxide generated during the transportation process is as follows:

2 $${\text{Y}} = {\text{F}}\times {\text{L}}\times {\text{N}}$$

where Y represents carbon emissions, F represents the emission coefficient of transport vehicle; L represents the transportation distance; N represents the amounts of materials.

The carbon emission coefficient method directly calculates the carbon dioxide emission by multiplying the carbon dioxide emission coefficient of a specific material by the amount of this material, which applies to the situation where the statistical data cannot be directly obtained from the enterprise^25^. In this study, this method was employed to calculate the carbon emissions generated during the production of building materials. Calculating carbon emission factors by experimental determination is complicated, and there has been a unified carbon emission factor database for building materials. Therefore, published carbon emission factor databases are frequently employed in most current researches^26–29^. Since the calculation results are more objective and accurate when employing the databases with the same source, the carbon emission factors of building materials in this research are all based on the carbon emissions list issued by the Department of Mechanical Engineering of Bath University, as shown in Appendix I (Stone Federation Great Britain, 2011). The list is calculated by SIS Tech (a sustainable development research institute in the UK) in cooperation with the Heriot-Watt University. And, the data source is precise and reliable, covering the concrete, granite, cement, and sand required in this study.

The corresponding calculation formula is:

3 $${\text{Q}}_{\text{2}} = {\text{N}}\times {\text{I}}_{\text{2}}$$

where Q_2_ is the amount of CO_2_ emitted by the production of materials; N is the number of materials; I_2_ refers to the carbon dioxide emission per unit of cement, natural sand, and stone.

#### Calculation of carbon emissions in the production stage

This stage includes carbon emissions generated during the extraction and production of the required raw materials. Based on carbon emission factor method, the calculation equation is as follows:

4 $${\text{C}}_{{\text{p}}} = \sum \begin{array}{*{20}c} n \\ {i = 1} \\ \end{array} ({\text{m}}_{{\text{i}}} \times {\text{g}}_{{\text{i}}} )$$

where C_p_ is the amount of carbon emission emitted by the production stage, n is the total number of material types, m_i_ is the amount of type ‘i’ (kg or m^3^); The value of mi represents the amount of material that is required (Paving area multiplied by pavement thickness), g_i_ is the carbon emission coefficient of type ‘i’(kg/kg or kg/m^3^ CO_2_).

#### Calculation of carbon emissions in the transportation stage

This phase refers to the transportation process from the origin to the construction site after completing the production of raw materials. During the transportation process, vehicles will consume a certain amount of fuel and release greenhouse gases such as carbon dioxide. The calculation formula is as follows:

5 $${\text{C}}_{{\text{t}}} = \sum \begin{array}{*{20}c} n \\ {i = 1} \\ \end{array} ({\text{f}} \times {\text{L}}\times {\text{N}}_{\text{i}} )$$

where C_t_ is the amount of carbon emission emitted during the transportation stage. f represents the emission coefficient of the corresponding vehicle in the transportation process; L refers to the transportation distance (km); N_i_ is the mass of material ‘i’ (t).

#### Calculation of carbon emissions in the construction stage

The fuel and electricity consumed by the construction machinery used in this stage will generate carbon emissions. The calculation equation is as follows:

6 $${\text{C}}_{{\text{c}}} = \sum \begin{array}{*{20}c} n \\ {i = 1} \\ \end{array} ({\text{Q}}_{{\text{i}}} \times {\text{R}}_{{\text{i}}}/ {\text{F}}_{\text{i}} )$$

where C_c_ is the amount of carbon emission emitted during the construction stage. Q_i_ refers to the energy consumption of machinery i during the construction; R_i_ refers to the carbon emission factor of the energy used by machinery ‘i’; F_i_ refers to the area that machinery ‘i’ can construct per hour. After the ground pavement project in this study is completed, the service life of the plaza could be 100–150 years. Furthermore, different construction methods have no discernible effect on the square's service life. As a result, the impact of lifespan on carbon emissions is not taken into account in this study.

## Case study analysis and results

### Life cycle carbon emissions of concrete ground paving

**Figure 2:**
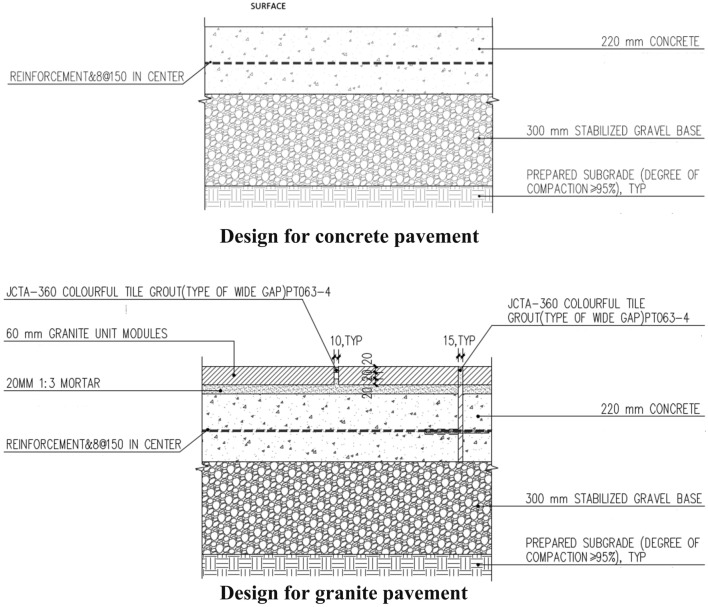
Structural design drawing of the ground paving of the site (a world-class theme park Plaza in Beijing). Design thickness based on the performance of different materials while ensuring ground functionality.

1. Production Stage: Fig. 2 displays that the primary raw materials used for CAC ground paving are concrete. Designers created different paving thicknesses based on various materials to ensure pavement functionality. The pavement per m^2^ is used as the unit in this study to investigate the differences in carbon emissions from various materials used in the same project. According to the structural design drawing, 519.2 kg of concrete is consumed per m^2^ of ground paving, and the total carbon emissions in the production phase are 67.496 kg/m^2^, according to Table 1.
2. Transportation Stage: this study investigated the production sites of cement and aggregate in three concrete batching plants and found that the average transportation distance of cement and aggregate from origin to the project site is 124 km and 129 km, respectively. According to the proportion of cement and aggregate in concrete, the comprehensive transportation distance of concrete is 128 km. The total carbon emissions in the transportation phase were calculated based on the transportation energy consumption of diesel vehicles, which is 7.77 kg/m^2^ (Table 2).
3. Construction Stage: the equipment used at the concrete pavement construction site is a concrete polisher, with an average construction rate of 200 m^2^/h. Its energy consumption is shown in Table 3. The total carbon emissions in this construction phase are calculated to be 0.1910 kg/m^2^.

**Table 1:** Calculation of the carbon emissions during the production stage.

Case	Materials	Amount (kg)	Carbon emission factor (kg CO_2_/t)	Carbon emissions (kg CO_2_/m^2^)	Stage amount (kg/m^2^)
Architectural concrete	General concrete	519.2	130	67.496	67.496
Granite	Granite	167.4	93	15.5682	88.378
	General concrete	519.2	130	15.5682	
	Cement	5.2	830	4.316	
	Sandstone	15.6	64	0.9984

**Table 2:** Calculation of the carbon emissions during the transportation stage.

Case	Materials	Vehicle	Amount (kg)	Distance (km)	CO_2_ eq^34^ (kg/t km)	Stage amount (kg CO_2_/m^2^)
Architectural concrete	General concrete	Lorry > 32t	519.2	128	0.117	7.77
Granite	Granite	Lorry > 32t	167.4	688	0.117	21.562
	General concrete Cement Sandstone	Lorry > 32t	540	128	0.117	

Note: The data of fuel energy consumption and carbon emission factors in the table are obtained from “IPCC Guidelines for National Greenhouse Gas Inventors” and Ecoinvent Life Cycle Inventory (LCI) data. And the emissions of production stage of the fuel were not included.

**Table 3:** Calculation of the carbon emissions during the construction stage.

Case	Equipment	Energy consumption^a^	Efficiency (m^2^/h)	Carbon emission factor (kg CO_2_ /unit))	Stage amount (kg CO_2_/m^2^)
Architectural concrete	Concrete polisher	23.14 kwh (Electricity)	200	0.950	0.1099
		7.24 kg/h (Gasoline)	200	2.240	0.0811
					Subtotal: 0.1910
Granite	Concrete mixer	15.54 kg/h (Diesel fuel)	50	2.701	0.8395
	Forklift	3.30 kg/h (Diesel fuel)	300	2.701	0.0297
					Subtotal: 0.8686

Note: Mechanical energy consumption data are obtained from the "National Carbon Emission Calculation Standard for Buildings" (GB/T51366-2019).

^a^The emissions of production stage of the fuel were not included.

It can be calculated that the total carbon emissions of the CAC ground paving over the life cycle are 75.457 kg/m^2^.

### Life cycle carbon emissions of granite ground paving

1. Production Stage: Fig. 2 displays that the primary materials used for granite ground paving in this study include 60 mm granite, 20 mm cement, and 220 mm concrete from top to bottom. 519.2 kg of concrete, 167.4 kg of granite, 5.2 kg of cement, and 15.6 kg of sandstone were used per m^2^ of the ground paving. The total carbon emissions in this stage were calculated to be 88.378 kg/m^2^, according to Table 1.
2. Transportation Stage: the transportation distance data for this study is provided by the case site construction team. When granite is used as a paving material, concrete and sandstone are still required, according to the construction drawings (Fig. 2). So, it’s important to not only think about the carbon emissions that happen when transporting granite, but also to figure out how much carbon dioxide is released when transporting general concrete and sandstone. The natural stone granite selected in this case was from the Stone Industrial Park in Jietou Town, Wulian County, Shandong Province, with a transportation distance of 688 km. The carbon emissions in the process were calculated based on the energy consumption of diesel vehicles. The transportation distance of concrete adopts the above comprehensive transportation distance of cement and aggregate from the origin to the project site. The total carbon emissions in this stage were calculated to be 21.562 kg/m^2^, according to Table 2.
3. Construction Stage: forklift and concrete mixer was used at the granite pavement construction site for the required 20 mm cement mortar, with an average output of concrete that can be paved with an area of 500m^2^ per hour. The total carbon emissions in this stage are 0.8686 kg/m^2^, according to Table 3.

It can be calculated that the total carbon emissions of the granite ground paving over the life cycle is 110.809 kg/m^2^.

### Comparative analysis of the carbon emissions of CAC and granite ground paving over the life cycle

**Figure 3:**
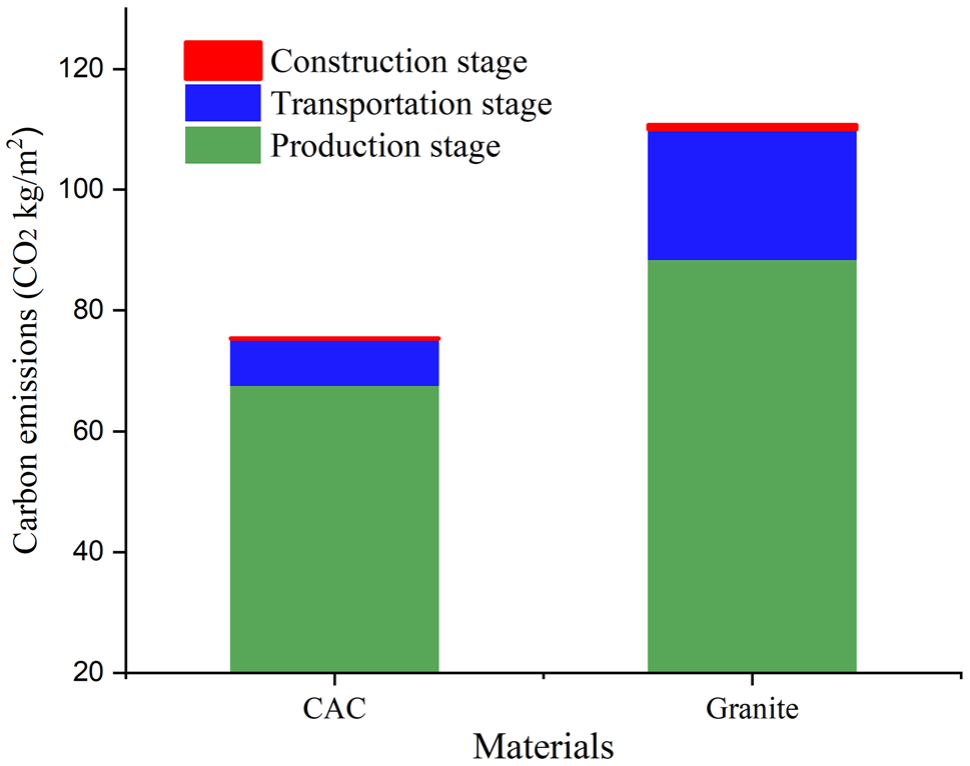
Comparison of total carbon emissions (kg CO_2_/m^2^) of cast-in-place architectural concrete (CAC) and granite ground paving of a world-class Theme Park Plaza in Beijing.

**Figure 4:**
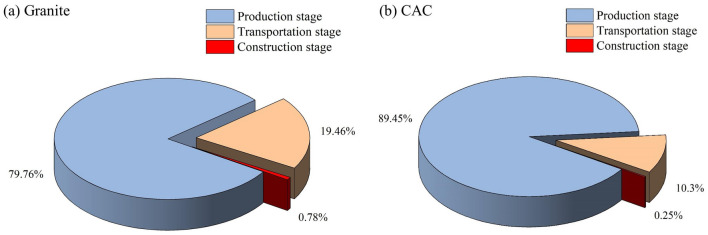
Carbon emissions at each stage of granite and cast-in-place architectural concrete (CAC) pavement of a world-class Theme Park Plaza in Beijing.

This research explores the carbon emissions intensity of different materials from the case study. Results show that different paving structures will cause differences in carbon emissions intensity. As illustrated in Fig. 3, the carbon emissions of the granite ground paving in this study are 110.81 kg CO_2_/m^2^, while that of CAC is 75.46 kg CO_2_/m^2^. Figure 4 demonstrate that in the life cycle of the two materials, the production phase accounts for the maximal part of total carbon emissions, followed by the transportation phases. Carbon emissions from production and transportation exceed 99% of total emissions. The carbon emissions in the construction stage account for a tiny proportion because large machinery like the crane tower was not used on construction location. The carbon emissions of granite pavement are higher than that of CAC, because concrete can be used as a structural layer with surface decoration. By contrast, granite can only serve as the surface decoration layer in the ground structure of granite pavement, and the cushion is a concrete layer with the same thickness as in the CAC ground. Therefore, the total carbon emissions of granite pavement also include the carbon emissions of granite and cement mortar compared with the CAC pavement. According to Manuel’s study, paving sidewalks with granite slabs results in 35 kg more CO_2_ emissions per m^2^ than with concrete slabs^20^. This is due to granite's excellent hardness and abrasion resistance, which necessitates the use of powerful machinery to cut it. As a result, a large amount of energy consumption will be generated when mining and processing granite. Additionally, CAC is widely accessible in many cities, whereas stone materials must be transported from far-off origins to the construction site. Therefore, the stone pavement will consume a large amount of fuel in the transportation stage, thus increasing carbon emissions.

## Analysis of carbon source and emission reduction potential in the ground paving of the plaza

### Influencing factors of carbon emissions in construction projects

The carbon emissions generated by granite pavement in Spain during the material production stage are very similar to our research^20^. However, a concrete motorway in Florida is higher than our study^19^. The density of materials used in different regions varies, and carbon emission factors from different sources are used, which explains the differences in carbon emissions during the manufacturing phase. In the future, it is critical to specify a standard carbon emission factor pool based on regional differences. In this study, the transportation carbon emission factor is 0.117 kg CO_2_ (t/km). This finding is consistent with the findings of numerous studies. Transportation data for recycled concrete and concrete pavement from China, for example, show 0.111 kg CO_2_ (t/km) and 0.1759 kg CO_2_ (t/km) emissions, respectively^30,31^. There is also a small gap between the multi-purpose university building in Sri Lanka and this study, which was 0.1526 kg CO_2_ (t/km)^5^. Because most modes of transportation use trucks, and some regions use concrete mixer trucks, but almost all use diesel fuel. At this stage, however, different transportation distances can lead to distinct total emissions^20^. Only about 1% of carbon emissions occur during the construction phase. A concrete mixer, a forklift and a troweling machine capable of constructing 200–500 m^2^ per hour were used in this study. When large machinery, such as bulldozers, road rollers, asphalt mixing equipment, and cranes, are used in construction, the carbon footprint increases^19^.

Compare with similar studies, Wu calculated the carbon emissions of recycled concrete pavement bricks across the whole life cycle, and the result was 317.53 kg/m^3^^14^. According to the thickness mentioned in the article, each m^3^ of concrete brick can pave 20 m^2^ of pavement, results of carbon emissions was 15.8 kg/m^2^. This result is much lower than our research due to the low material consumption, thin thickness, and short transportation distance of recycled concrete pavement bricks. Moreover, the life cycle of Wu's study included the production of natural and recycled aggregates, and the demolition of waste concrete. Additionally, the carbon emissions of granite pavement in this study is far higher than that of Suzhou tennis court (5.39 kg/m^2^)^8^. The reason is that the recycling stage in the life cycle of granite was included in that study, which partially offset carbon emissions, and since the construction site was unique, it was not necessary to estimate the carbon emissions of transporting the building materials from the production workshop to the construction site. However, the results of this study are close to the carbon emissions (110 kg/m^2^) of granite pavements in Spain^20^. As displayed in Table 4, the consequences of this research are far below those of projects such as grassland wind farms^40^, prefabricated floor slabs, composite beams^33^, and others because there were fewer building materials used in this study, no need for large-scale equipment during construction, and manual cleaning rather than mechanical maintenance was used to maintain the square while it was in use. However, compared with residential^10^, wood construction^34^, fill dams^17^, and other projects, the carbon emissions in this study are higher because it is more environmentally friendly by selecting appropriate materials according to the function and service life of the construction.

**Table 4:** Carbon emissions analysis of architecture projects through life cycle assessment.

Project type	Main materials	Carbon emissions (kg CO_2_)	Units	City	References
Fill dams	Soil and rock	0.16–7.38	m^3^	South Korea	^1^
Multi-purpose University building	Ready-mixed concrete, reinforcement steel, Clay bricks, random rubble, cement, sand	31.38	m^2^/year	Sri Lankan	^5^
Granite of garden Pavement	Granite	5.39	m^2^	Nanjing	^8^
Residential	Steel, cement	28.10	m^2^/year	Tianjin	^10^
Recycled Concrete Pavement Brick	Concrete	317.53	m^3^	Yangzhou	^14^
Grassland wind farms	Steel, copper, cast iron, epoxy resin	623	m^2^	Inner Mongolia	^40^
Prefabricated floor Slab	Concrete, rebar	578.746	m^3^	Shaoxing	^32^
Composite beams	Reinforced bars and concrete	1045	m^3^	Fujian	^33^
Wooden construction	Timber Reinforced concrete	60.2	m^2^	Taiwan	^34^
Sidewalks	Granite	110	m^2^	Barcelona	^20^

In conclusion, the carbon emissions of construction projects were mainly affected by material selection, transportation distance, and construction machinery. In addition, the difference in the definition of life cycle time boundary has a considerable effect on carbon emissions as the basis and premise for the calculation. Liu's research found that when the life cycle boundary is different, the change rate of carbon emissions will be as high as 70%^40^. This conclusion has also been confirmed in the life cycle assessment of the urban^35^ and farmland ecosystems^36^. If our study employs a cradle-to-grave life cycle, with the carbon emissions of the recycling stage included, the calculation results will change unpredictably. However, the carbon emissions from the recycling stage were not factored into the calculation, because the research site in this study is Theme Park Plaza, whose ground is only for people, not heavy trucks, and the business period of this research site is quite lengthy.

### Model construction and analysis of more cases

After analysis and calculation, the results show that the carbon emissions generated in the production and transportation phases of the ground paving process account for more than 99% of the total. As a result, the carbon emissions model in this research will disregard the construction stage. There are almost no vehicles on the square, which is primarily used as a sidewalk. As a result, the plaza’s daily management consists primarily of manual cleaning, which does not necessitate extensive mechanical maintenance. Furthermore, the paving material, whether concrete or natural stone, can last for more than 100 years. Accordingly, the maintenance and recovery phases are not considered in the model construction. In summary, the carbon emissions calculation model developed in this study for ground paving is as follows:

7 $${\text{C}} = \sum\nolimits_{i = 1}^{n} {\left( {{\text{mi}} \times {\text{gi}}} \right)} + \sum\nolimits_{i = 1}^{n} {({\text{f}} \times {\text{L}} \times {\text{N}}_{{\text{i}}} )}$$

where C is the amount of carbon emission, n is the total number of material types, mi is the amount of type ‘i’, gi is the carbon emission coefficient of type ‘i’. f represents the emission coefficient of the vehicle in the transportation process; L refers to the transportation distance; Ni is the mass of material ‘i’.

In order to more accurately analyze the determinants of carbon emissions from plaza ground paving projects, in addition to concrete (A) and granite (B), this study assumes five cases with reference to the carbon emission factors and densities of marble (C), clay tiles (D), slate (E), sandstone (F) and finish tiles (G). The transportation distance is set randomly and the carbon emissions are calculated by substituting into the above model. And the thickness of the material laying was 60 mm concerning the design drawings of this study. The results are listed in Table S1.

### Sensitivity analysis of influencing factors

**Figure 5:**
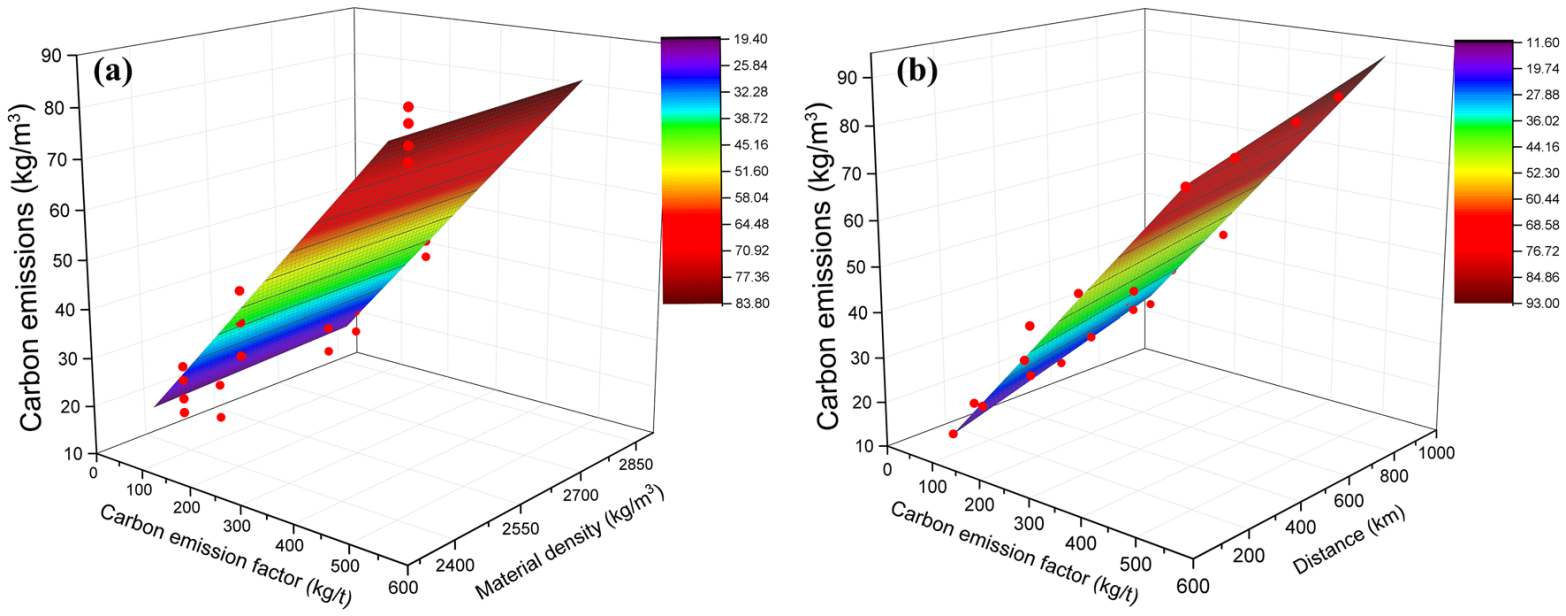
Fitting plane between carbon emission factor, material density, transport distance with carbon emissions of plaza ground paving. (Result of ‘(**a**)’ obtained by uniformly setting the distance to 300 km, result of ‘(**b**)’ obtained by uniformly setting the density to 2500 kg/m^3^).

The above equation shows that the factors influencing total carbon emissions include carbon emission factor, transportation distance, and material density. Therefore, in this study, surface models were constructed to reflect the relationship between carbon emissions and the three variables when the transportation distance and material density were constant, respectively (Fig. 5).

Grey relation analysis is a technique to assess the level of correlation between variables ground on their trend similarity or dissimilarity. The basic idea is to evaluate the stiffness of the correlation ground on the geometric shape of the sequence curve^37^. The grey relational correlations of carbon emissions with material carbon emission factor, material density, and transportation distance were calculated to be 0.790, 0.724, and 0.662, respectively (Table 5). A grey correlation coefficient above 0.5 suggests the presence of a linkage between both the two, and the larger the coefficient, the stronger the correlation^38^. As a result, there is a relationship between the life cycle carbon emissions of ground paving and the material carbon emission factor, material density, and transportation distance, with the material carbon emission factor having the highest sensitivity, followed by the material density, and finally the transportation distance.

**Table 5:** Grey correlation analysis results.

Evaluation item	Grey relational coefficients	Ranking
Carbon emission factor	0.790	1
Material density	0.724	2
Distance	0.662	3

**Figure 6:**
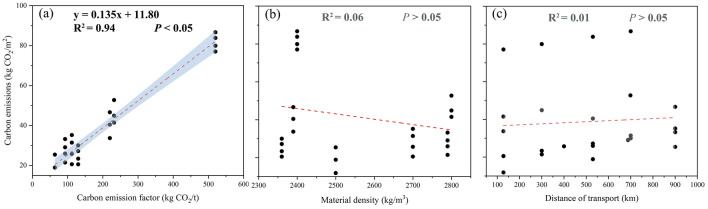
The linear relationship between total carbon emissions from plaza ground paving and material carbon emission factor, material density, distance of transport.

Furthermore, to gain a greater appreciation for the connection between carbon emissions and material density, transportation distance, and material carbon emission factors, this study used Pearson correlation analysis to fit the above relationships separately. The carbon emission factor of materials is significantly correlated positively with carbon emissions, according to Fig. 6. However, Pearson’s analysis did not find a linear relationship between transportation distance and material density.

### Carbon emissions reduction potential in ground paving

To summarize, the consumption of electricity or oil for large machinery in the material production phase is the main carbon source in the ground paving project, followed by the consumption of oil trucks during material transportation from the origin to the construction site. The key factor affecting carbon emissions is the carbon emission factor of materials, followed by the density and transportation distance of materials. As a result, the carbon reduction of the ground paving project should concentrate on material selection and energy-efficient transportation.

The stone mining industry has problems of environmental damage and resource waste that cannot be ignored^39^. In the background of promoting the construction of the low-carbon city, sponge cities and ecological cities, the transformation of the stone industry is a vital measure to saving energy and lowering emissions when facing climate change. To meet the goal of low carbon emission and not affect performance, it is critical to select materials with low carbon emission factors. The material production stage can also cut emissions by optimizing the production process and increasing the material resource utilization.

Numerous studies demonstrate that the plurality of carbon emissions in the building engineering sector are induced during the phases of material production and transportation^14^. To limit greenhouse gas emissions, it is critical to select materials with close origins without compromising building functions. Similarly, since different fuels (diesel, engine oil, electricity) or vehicles with varying specifications affect carbon emissions, it is crucial to choose an energy-efficient method of transportation^33^. Promotion of construction management and green building practices is also required in the construction stage^29^. For instance, choosing more ecofriendly fuels for the heavy equipment used in construction will help to lower greenhouse gas emissions. Reduction of emissions during the installation and construction phase can also be achieved by improving the construction plan.

## Conclusion

1. In this research, the life cycle system boundary of the piazza ground paving project was determined and a carbon emissions calculation model was created. The carbon emission of cast-in-place architectural concrete is lower than that of natural stone granite ground paving under the premise of the same use function, according to the results of carbon emissions calculation of two kinds of materials for ground paving of a world-class Theme Park Plaza in Beijing, which was 75.46 kg CO_2_/m^2^ and 110.81 kg CO_2_/m^2^ respectively.
2. In the life cycle of ground paving, the carbon emissions are primarily caused by the manufacturing stages of the construction materials, with the construction stage taking into consideration less than 1% of the total. The main carbon source in the piazza ground paving project is electricity or oil for large machinery in material production, followed by oil trucks during material transportation from the origin to the construction site.
3. The key factor affecting carbon emissions is the carbon emission factor of materials, followed by the density and transportation distance of materials. The carbon emissions factor of materials and the carbon emissions of engineering have a significant linear relationship.
4. In summary, it can be concluded that utilizing environmentally friendly and locally sourced building materials and cleaner fuels can shorten the transportation distance and effectively reduce the greenhouse gas in the process of ground paving of the park plaza.

## Limitations and future studies

Some limitations in carrying out this study could be recognized. For starters, basic data on carbon emission factors in China has remained deficient. Some variables could only be acquired through data collected in other countries. Second, the study area for this research was a large park in Beijing. The findings may not be directly applicable to other locations. Furthermore, due to a scarcity of real projects data sources, there are limitations in the construction of the surface model and the analysis of the Pearson correlation. In the future, we still need to collect multiple real projects data to verify the model of this study. Even so, the research results may contribute to the applicable body of knowledge. The findings might offer a scientific foundation for better construction management and the promotion of low-carbon building technology. They might serve as a guide for by administration and developers in developing and refining rules and regulations for conservation of energy and reducing emissions. Future work could assess ground paving carbon footprint in other origins in order to develop an accurate carbon emissions calculation agreement for numerous states.

## Data Availability

The datasets during the current study are available from the corresponding author on reasonable request.

## Appendix I

**Table:** Carbon emission factors of common building materials.

Building materials	Carbon emission factors (Kg CO_2_/t)
Sandstone	64
Granite	93
Marble	112
General Concrete	130
General Clay Bricks	220
Slate	232
Timber	450
Facing Bricks	520
General Building Cement	830
Steel: Bar and Rod	1710
Steel: Galvanized sheet	2820

Note: source: Natural Stone-The Oldest Sustainable Materialand & University of Bath ICE.

## Appendix II

**Table:** Abbreviations.

Full name of the term	Abbreviations
Life cycle assessment	LCA
Cast-in-place architectural concrete	CAC
Intergovernmental Panel on climate change	IPCC
The carbon emission in the stage of the life cycle stage	C_T_
The carbon emission in the stage of the raw material production	Cp
The carbon emission in the stage of the raw material transportation	Ct
The carbon emission in the stage of construction of pavement	Cc
Carbon emissions at a certain stage	Y
